# Preventive Effects of Aqueous Extract of *Berberis integerrima *Bge. Root on Liver Injury Induced by Diabetes Mellitus (Type 1) in Rats

**Published:** 2015

**Authors:** Hossein Ashraf, Samad Zare

**Affiliations:** *Department of Biology, Faculty of Science, Urmia University, Urmia, Iran.*

**Keywords:** *Berberis integrrima*, Diabetes mellitus, Hepato protective, Antioxidant, Rats

## Abstract

This study was conducted to assessthe preventive effect of aqueous extract of *Berberis integerrima* Bge. root (AEBIR) on liver damage and oxidative stress induced by diabetes mellitus in rats. Forty male rats were divided into 5 groups as follows: 1- normal (N); 2- normal + barberry (N+B) (they received barberry root extract for 6 weeks); 3- diabetic (D) (they received Streptozotocin (STZ), 65 mg/Kg BW /*i.p*.); 4- diabetic +barberry before (D+Bb) (they received barberry root extract for 3 weeks before STZ injection and continued for another three weeks); and 5- diabetic + barberry after (D+Ba) (three days after STZ injection, they received barberry root extract for 3 weeks). The experimental groups received barberry root extract (500 mg/Kg bw) intra gastric by gavage for 6 weeks. The treatment of diabetic rats with AEBIR showed a significant decreases(p<0.001) in levels of blood glucose, malondialdehyde (MDA), alanine aminotransferase (ALT), aspartate aminotransferase (AST), alkaline phosphatase (ALP) and total bilirubin while body weight, total protein, superoxide dismutase (SOD), catalase(CAT) and reduced glutathione (GSH) increased (p<0.001) in comparison to diabetic control rats. Consumption of AEBIR in group D+Bb caused significant improvement in all these factors, compared to the group D+Ba. Also in this study, for the first time, we demonstrated that administration of AEBIR before diabetes induction resulted in enhanced amelioration of liver complications compared to the group receiving it after induction, indicating that AEBIR can play a preventive role in such patients.

## Introduction

Diabetes is a chronic, lifelong disease and one of the most common endocrine disease, that most of the time cause due to reduced insulin secretion by beta cells (1). The disease is an increasingly prevalent metabolic disorder in humans and is characterized by hyperglycemia (2). This is due to either a lack of insulin or insensitivity of insulin to target cells (3). Diabetes can be divided primarily into two types: Type 1 or insulin dependent diabetes and type 2 or non- insulin dependent diabetes (2). Type 1diabetes is due to autoimmune destruction of β-cells, especially in childhood. On the other hand, type 2 diabetes is mainly due to hereditary factors, affluent lifestyles and obesity (2). Both types of diabetes are associated with a number of common symptoms such as polyurea and polydipsia, and long term complications including retinopathy, cardiomyopathy, nephropathy and digestive insufficiency, especially if the disease is not diagnosed and treated early (2, 50). Both type 1 and type 2 diabetes damage a variety of ocular tissues (51, 52). Liver is an important organ in maintaining concentrations of blood glucosewithin a narrow, normal range, and increase in blood glucose may lead to imbalances in oxidation-reduction reactions in hepatocytes (4). Several studies have indicated that streptozotocin has adverse effects on the liver and kidney tissues (5,6). Furthermore, the role of free radicals in streptozotocin-induced tissue damages in diabetic rats has been demonstrated (7). Scince regards diabetes can cause the multiple and sometimes fatal disorders in people with diabetes, it is necessary to investigate the ways to treat, prevent and reduce the incidence diabetes. In synthetic drugs that lower the blood sugar level are unable to control tissue damage in diabetes, they can also cause many side effects (8). Todays Plants play an important role in synthesizing drugs (9). Antioxidants founded in food and the body, even in small amounts, can protect the body against oxidative stress-induced free radicals (10). This study was performed on *Berberis integerrima *Bge. (Syn: *Berberis densiflora* Boiss. &Buhse). This plant belongs to the *Berberidaceae* and has too much medicinal properties (11, 12, 53, 19). Various properties are listed for different parts of barberry, in addition to the effects of antioxidant in barberry fruit (13), root and stem bark contain various alkaloids, among which *berberine* is the most important one (14). On Barberry root extract and its main alkaloid (*Berberine*) the following properties are listed: antioxidant and collecting free radicals (15), anti-inflammatory (14), hypoglycemic andhypolipidemic (16), renal protective (6), hepatoprotective (17) and etc properties.

Given that antioxidant, anticancer (18), hepatoprotective (19), hypoglycemic and hypolipidemic (16) properties of properties of *Berberis integerrima* Bge*. *have been demonstrated, and since all recent studies has focused on therapeutic role of barberry in diabetes, we decided to assessthe preventive effect of AEBIRon liver injury and antioxidant system in streptozotocin-induced diabetic rats.

## Experimental

*Plant collection and extraction*

Wild samples of barberry roots were collected from the outskirt, Bavanat (Fars, Province, Iran). A specimen of plant was submitted at herbarium of faculty of science of urmia university (Iran), and was identified by the botany department, and was impounded in the herbarium (No. 9059). Roots after washing with cold water were dried in the shade then were powdered by using mechanical grinding. The aqueous extract was prepared by cold maceration of 150 g of powdered root barks in 500 mL of distilled water for 72 h. Then the extract was filtered through a Whatman No.1 filter paper to obtain a clear extract. The filtrate was concentrated by water bath (65 °C) for 48 h, dried in vacuum (yield 10 g) and the residue was stored in a refrigerator at 2-8 °C for use in subsequent experiments (20). The required concentration was prepared in accordance mg/Kg body weight by normal saline.

*Animals*

Male Wistar rats weighing 220-250 g were obtained from the Pasteur Institute central animal house (Tehran, Iran) and were housed in an air-conditioned room under a 12 h light-dark cycle. The animals were given water ad libitum and fed with standard laboratory diet, after randomisation into various groups, the rats were acclimatized for a period of 6-7 days in another environment before starting the test. Animals described as fasted were deprived of food for at least 12 h, but had free access to water. The study was approved by the Institution’s Ethical Committee.

*Acute toxicity study*

The acute toxicity study for aqueous extract of *Berberis integerrima *Bge. was performed using Wistar rats according to the acute toxic classic method as per the OECD guideline No. 423 (Acute Toxic Class Method). The animals were fasted overnight prior to the experiment and maintained under standard conditions. aqueous extract of *Berberis integerrima *Bge. was found safe up to dose of 2,500 mg/Kg of body weight. The rats were observed continuously for 24 h for behavioral, neurological and then at 24 h and 72 h for any lethality (21). Hence 1/5^th ^(500 mg/Kg) of this dose were selected for further study. The selected dose of the extract was based on initial tests.

*Streptozotocin-induced diabetic rats*

Fasted Wistar rats were intraperitoneal injected with 65 mg/Kg bw, dissolved in 0.1 cold citrate buffer (ph=4.5) (47) of streptozotocin (Sigma chemical Co (St Louis, Mo, USA), while non-diabetic rats was injected with citrate buffer alone without streptozotocin by the same route. After 72 h of streptozotocin administration, the serum glucose was measured by ACCU-Check glucose meter and animals were considered as diabetic when the observed glucose level was above 300 mg/dl (22).

*Experimental design*

The animals were randomly divided into 5 groups (n=8). Group 1: Normal control rats received normal saline; Group 2: Normal control treated rats received 500 mg/Kg/day of AEBIR; Group 3: diabetic control rats received normal saline; Group 4: diabetic + barberry before (D+Bb), received barberry root extract (500 mg/Kg/day) for 3 weeks before STZ injection and continued for another three weeks and Group 5: diabetic + barberry after (D+Ba) three days after STZ injection, they received barberry root extract (500 mg/Kg/day) for 3 weeks. Animals were treated daily by gavage for 6 weeks and the experimental period for each rat was 6 weeks (16).

*Blood collection*

At the end of 6 weeks, 24 h of the last treatment, all the animals were anesthesized in a chloroform (Pharmaceutical Partners of Japan) chamber. Blood was collected from animal's hearts by heparinized syringes and kept at 37 °C for 30 minutes. Serum obtained by blood centrifugation (3000 rpm at 4 ^0^C for 15 min) and stored at -30 °C for different biochemical analysis.

*Estimation of blood glucose*

Blood samples were collected for estimating fasting blood glucose before the study, and at 3 and 6 weeks after the study on lateral tail vein, by using an ACCU-Check glucose meter (Roche, Mannheim, Germany).

*Estimation of body weight*

The body weights in experimental animals were determined before the study, and at 3 and 6 weeks after the study by digital balance. These weights were determined at the same time during the morning.

*Estimation of some serum biochemical parameters*

The biochemical parameters like serum enzymes: alanine aminotransferase (ALT), alkaline phosphatase (ALP), aspartate aminotransferase (AST), total bilirubin, total protein and albumin determined with the use of commercially available enzyme kits (Pars Azmoon, Tehran, Iran), according to the methods described by the manufactures and using an automatic analyzer (Architect c8000 Clinical Chemistry System, USA).

*Estimation of lipid peroxidation and antioxidant enzymes*

The dissected livers were washed with normal saline and hemogenated (10%) in ice-cold phosphate buffer and centrifuged at 12,000 × g for 30 min at 4 °C. The supernatant was collected and used for enzymatic studies. Lipid peroxidation assay (MDA) was carried out by the modified method of Wills (23), Superoxide dismutase (SOD) activity by the method of Kakkar *et al.* (24) ^,^the activity of Catalase (CAT) by the method of Gott (25) and reduced glutathione (GSH) by the method of Ellman (26) were estimated.

*Histopathological studies*

Another piece of liver tissue were washed with normal saline and processed for histopathological observation. livers were fixed in 10% formalin and processed by the usual method for paraffin embedding, sections of 5 µm thickness were taken, stained with hematoxylin and eosin (H&E).

*Statistical Analysis*

All the data were expressed as mean ± SEM (Standard Error Mean). Statistical analysis was performed by one-way ANOVA followed by Turkey’s multiple comparison tests. Differences between groups were considered significant at p<0.05 levels.

## Results

*Body weight*

As shown in Table 1, normal control animals were found to be stable in their body weight. In STZ-induced diabetic rats the body weight were significantly decreased (p<0.001) in comparison to their normal rats during 6 weeks. Administration 500 mg/Kg BW of AEBIR in diabetic treated groups (D+Bb and D+Ba) lead to significant decrease (p<0.001) in body weight as compared with untreated STZ-induced diabetic rats in 6 weeks. Not significant difference was observed between D+Bb and D+Ba groups.

*Blood glucose*

The hypoglycaemic effect of AEBIR on the fasting blood sugar levels of normal and diabetic rats is shown in Table 1.In STZ-induced diabetic rats the fasting blood glucose levels were significantly increased (p<0.001) in comparison to their normal levels. Administration 500 mg/Kg BW of AEBIR in diabetic treated groups (D+Bb and D+Ba) lead to significant decrease (p<0.001) in serum glucose level as compared with untreated STZ-induced diabetic rats in 6 week. Also, a significant decrease (p<0.05) was observed in serum glucose level in D+Bb group than the D+Ba group in 6 weeks.

**Table 1:** Effect of AEBIR on the Blood glucose and body weight in normal and diabetic rats.

**Group** **(n=6)**	**Treatment**	**Dose** **(mg/kg)**	**Blood glucose level ** **(mg/dl)**			**Average** ** body weight** ** (g)**		
			**Week 0**	**Week 3**	**Week 6**	**Week 0**	**Week 3**	**Week 6**
1	**N+C**	10 ml/kg	92.8±6.1	86.8±3.6	91.6±4.5	243.6±15.1	261±8.4	290.8±5.9^&^
2	**N+B**	500	90.4±5.2	81.9±4.	80.4±3.8	231.6±6.6	254±6.4	278.4±6.4^&^
3	**D+C**	10 ml/kg	80.8±5.4	291.8±8.9^#^	305.5±10.1^#&^	230.2±6.2	180±8.8^#&^	150.4±6.75^#&^
4	**D+Bb**	500	98.5±4.4	81.2±8.9*	96.4±4.2*	239.6±6.2	255±7.6*	258.2±6.3*
5	**D+Ba**	500	82.6±5.2	90.6±10.1*	123.8±7.1* ^a&^	239.6±5.9	258±12.4*	241.4±10.3*

D+Bb : Diabetic rats treated + barberry_ before. D+Ba : Diabetic rats + Barberry_ After.;Nnormal;Ccontrol;B barberry: D diabetic; Values are presented as mean ± S.E.M..; n = 8 in each group. One way ANOVA followed by tukeytest;

# p<0.001: Diabetic control rats were compared with Normal control Rats on corresponding day,

* p<0.001 Diabetic treated Rats were compared with Diabetic control Rats,

a p<0.01: Diabetic rats + Barberry_ After were compared with Diabetic rats treated + barberry_ before and

& p<0.001 compared to 0 value.

*Liver parameters*

Table 2 shows the mean values of AST, ALT, ALP activities and serum total bilirubin, total protein levels of both control and experimental groups after 6 weeks. In STZ-induced diabetic rats the activities of blood AST , ALT, ALP and the serum total bilirubin level were significantly increased (p<0.001), but serum total protein level was decreased (p<0.001) compared to their normal levels. Treatment of the STZ-induced diabetic rats by 500 mg/Kg BW of AEBIR in diabetic treated groups (D+Bb and D+Ba) lead to significant decrease in AST, ALT, ALP, total bilirubin and significant increase in total protein (only in D+Bb group) level as compared with untreated STZ-induced diabetic rats. Also, a significant decrease (p<0.05 for ALT and ALP, p<0.01 for AST and total bilirubin) and a significant increase (p<0.01 for total protein) in these parameters were observed in D+Bb group than the D+Ba group. 

**Table 2:** Effect of AEBIR on the liver parameters (mg/dl) in normal and diabetic rats.

**Group** **(n=8)**	**Treatment**	**Dose** **(mg/kg)**	**liver parameters ** **(mg/dl)**				
			**ALP**	**ALT**	**AST**	**Total bilirubin**	**Total protein**
1	**N+C**	10 ml/kg	99.08±6.17	52.62±3.08	39.86±4.97	1.59±0.13	7.51±0.41
2	**N+B**	500	91.42±2.17	50.18±5.01	35.18±5.09	1.30±0.11	7.53±0.44
3	**D+C**	10 ml/kg	178.81±6.40^#^	99.88±4.84^#^	94.18±4.42^#^	3.71±0.16^#^	3.97±0.28^#^
4	**D+Bb**	500	95.10±5.20^c^	46.05±3.55^c^	35.58±4.40^c^	1.81±0.11^c^	5.70±0.29^a^
5	**D+Ba**	500	127.76±9.58^c*^	66.91±3.91^c^*	65.40±6.50^b^**	2.74±0.19^b^**	3.86±0.26*

D+Bb : Diabetic rats treated + barberry_ before. D+Ba : Diabetic rats + Barberry_ After.;Nnormal;Ccontrol;B barberry: D diabetic; Values are presented as mean ± S.E.M..; n = 8 in each group. One way ANOVA followed by tukeytest;.

# p<0.001 Diabetic control Rats were compared with Normal control Rats**. **

a p<0.05 and

b p<0.01,

c p<0.001 Diabetic treated Rats were compared with Diabetic control Rats;

* p<0.05 and

** p<0.01 : Diabetic rats + Barberry_ After were compared with Diabetic rats treated + barberry_ before.

*Antioxidant parameters*

Table 3 shows the mean values of SOD, CAT, GSH and MDA activities of both control and experimental groups after 6 weeks. In STZ-induced diabetic rats the activities of SOD, CAT and GSH were significantly decreased (p<0.001), but MDA increased (p<0.001) compared to their normal levels. However, all these parameters except MDA increased significantly in the diabetic groups treated 500 mg/Kg BW of AEBIR in diabetic treated groups (D+Bb and D+Ba) lead to significant increase in SOD, CAT, GSH and a significant decrease in MDA as compared with untreated STZ-induced diabetic rats. Also, a significant increase (p<0.05 for SOD, p<0.05 for CAT and p<0.01 for GSH) and a significant decrease (p<0.01 for MDA) in these parameters were observed inD+Bb group than theD+Ba group.

**Table 3:** Effect of AEBIR on antioxidant parameters in normal and diabetic rats.

Group (n=8)	Treatment	Dose (mg/kg)	MDA (nmol/g tissue)	SOD (u/mg tissue)	CAT (u/min)	GSH (nmol/g tissue)
1	N+C	10 mL/Kg	2.15±0.22	10.04±0.68	82.39±5.05	5.46±0.33
2	N+B	500	2.22±0.10	10.87±1.14	92.44±4.85	5.93±0.40
3	D+C	10 mL/Kg	7.43±0.59^#^	3.06±0.40^#^	32.64±3.31^#^	2.14±0.16^#^
4	D+Bb	500	2.23±0.32^c^	9.74±0.46^c^	86.59±5.57^c^	7.28±0.43^c^
5	D+Ba	50	4.48±0.45^c^**	6.32±0.61^a^*	57.63±8.45^ a^*	4.69±0.63^b^**

D+Bb : Diabetic rats treated + barberry_ before. D+Ba : Diabetic rats + Barberry_ After.;Nnormal;Ccontrol;B barberry: D diabetic; Values are presented as mean ± S.E.M..; n = 8 in each group. One way ANOVA followed by tukeytest;.

# p<0.001 Diabetic control Rats were compared with Normal control Rats**. **

a p<0.05 and

b p<0.01,

c p<0.001 Diabetic treated Rats were compared with Diabetic control Rats;

* p<0.05 and

** p<0.01 : Diabetic rats + Barberry_ After were compared with Diabetic rats treated + barberry_ before.

*Histopathology of liver*

**Figure 1:**
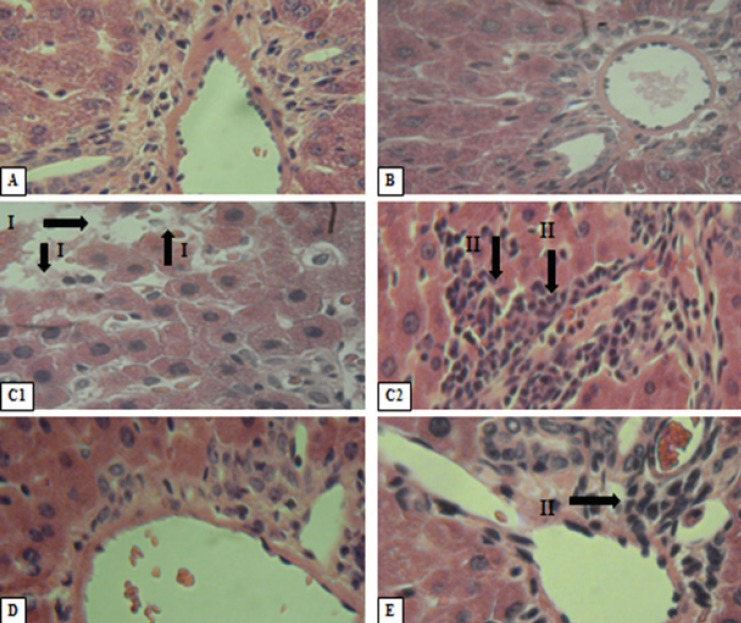
Histopathologicalevalution of liver sections. Formalin fixed liver sections of 5 μ thickness fromcontrol and diabetic were stained with H&E and images were taken at the magnification of 400X. Various panelsrepresent control (A), treated control (B), diabetic control (C1, C2), diabetic treated rats [D+Bb group](D) and diabetic treated rats [D+Ba group] (E).Total loss of hepatic architecture (I) and lymphocytic inflammation (II).

The liver (Figure 1) showed normal hepatic cells with well preserved cytoplasm, nucleus and central vein in normal control group. In diabetic control group, liver sections showed total loss of hepatic architecture, lymphocytic inflammation and focal necrosis of hepatic cells. In the D+Bb and D+Ba groups these changes improved before and after treatment with AEBIR (500 mg/Kg bw). However, total loss of hepatic architecture and partially lymphocytic inflammation was observed in D+Ba group. Overall, status of liver tissues in the D+Bb group were better than the D+Ba group and had almost returned to the normal level.

## Discussion

The present research was aimed to study the preventive and therapeutic effect effects of aqueous extract of *Berberis integerrima *Bunge. on liver injuryin STZ-induced diabetic rats. The results of this study revealed that AEBIR at the dose of 500 mg/Kg b.w. (specially in the D+Bb group) significantly normalized the elevated blood glucose level and restored serum and liver biochemical parameters towards normal values.

Streptozotocin (STZ) is drugs that possess diabetogenic properties mediated by pancreatic β cell destruction, hence this compound has been widely used to induce diabetes mellitus in experimental animals (27). Previous studies have demonstrated the hypoglycemic action of the extract of herbal plants in diabetic rats may be possible through the insulin mimictic action or by preventing the death of β cells and it may also permit recovery of partially destroyed β cells or by other mechanism such as stimulation of glucose uptake by peripheral tissue, inhibition of endogenous glucose production or activation of gluconeogenesis in liver and muscles (28). Diabetic rates treated by AEBIR showed a significant decrease in blood glucose level during 6 weeks of treatment in comparison with diabetic control. The activity of this fraction could be due to the presence of berberine and alkaloids components (14). Berberine may act as an *α*-glucosidase inhibitor, which is its main mechanism in diabetes treatment. The main mechanism of berberine in diabetes treatment may be act as the *α*-glucosidase inhibitor. The inhibitory effect of berberine on diabetes also might be associated with its hypoglycemic effect, modulating lipids metabolic effects and its ability to scavenge free radicals (48). However, the inhibition of intestinal glucose absorption or stimulation of peripheral glucose uptake also could be the another mechanisms of hypoglycemic effect of berberine (49). The maximum reduction in serum glucose levels was seen in D+B_b_ group. Hence, we could say that AEBIR had a beneficial effect on carbohydrate. AEBIR also showed marked effect in controlling the loss of body weight of diabetic rats.

Metabolic changes in the liver, such as administration of toxin, cirrhosis of the liver, hepatitis, liver cancer and diabetes cause a significant increase in AST and ALT enzymes (29). Similarly we observed in this study which the levels of AST and ALT in STZ- induced diabetes in rats were elevated. These enzymes may leak from the hepatocytes into the circulation where their levels become elevated (30). Therefore, the elevated level of AST and ALT in serum of STZ-induced diabetic rats suggests hepatocellular damage (31). Serum ALP, bilirubin and total protein level on the other hand are related to the function of hepatic cells. Increase in serum level of ALP is due to increased synthesis, in presence of increasing biliary pressure (30). Decline in serum total protein may be due to the inhibited oxidative phosphorylation processes which leads to decrease in protein synthesis, increase in the catabolic processes and reduction of protein absorption (32). In this study, AEBIR regulated the activity of ALT, AST, ALP, and bilirubin and total protein level in serum of rats intoxicated with STZ. These results werein accordance with the effect of *Berberis aristata* in diabetic rats. Furthermore, the beneficial effect of AEBIR in the D+Bb group on these parameters (ALT, AST, ALP, and bilirubin and total protein) was more than D+Ba group. 

Oxidative stress in diabetes mellitus has been shown to co-exist with a reduction in the endogenous antioxidant status (33). Several evidences suggest that STZ induces oxidative stress (34,35). Also oxidative stress was accelerated in diabetes mellitus owing to an increase in the production of oxygen free radicals and lipid peroxidation have been documented (36). Free radicals caused the mitochondrial enzyme damage and DNA breaks by intracellularly diffusion and subsequently following it caused the impairment of cellular function and contribute to the pathophysiology of diabetes (37). Also free radicals can stimulate the LPO that causes and damage to the cell membrane resulting in the formation of MDA, which the level of MDA reflects the degree of oxidation in the body (37). The treatment with AEBIR reduced the level of lipid peroxides indicating the effective antioxidant property of the AEBIR drug in the inhibition in free radicals (ROS) generation and moderation of tissue damage. Glutathione plays an important role in the endogenous non-enzymatic antioxidant system. Primarily it acts as reducing agent and detoxifies hydrogen peroxide in presence of an enzyme glutathione peroxidase (38). The lowered GSH level may be due to reduction in GSH synthesis or degradation of GSH by oxidative stress in STZ-induced hyperglycemic animals (39). AEBIR treatment significantly elevated the reduced hepatic glutathione levels towards normal in diabetic rats. The results showed that the antihyperglycemic activity of AEBIR was accompanied with the enhancement in non-enzymatic antioxidant protection. These findings suggest that the AEBIR may exert its antidiabetic effect through the enhancement of cellular antioxidant system. Enzymatic antioxidants (SOD and CAT) form the first line of the antioxidant defense mechanism to protect the organism from ROS mediated oxidative damage (40). The decreased activities of SOD and CAT may be a response to increased production of H_2_O_2_ and O_2_^-^ by the auto-oxidation of the excess of glucose and nonenzymatic glycation of proteins (41). In the present study, SOD and CAT showed lower activities in liver during diabetes and the results agree well with the earlier published data (42,43). AEBIR treatment significantly elevated the reduced hepatic SOD and CAT levels towards normal in diabetic rats. Alsothe use of AEBIR before injection of STZ (D+B_b)_ caused a significant increase in SOD, CAT and GSH activities and decreased the level of MDA in this group than the group that received AEBIR after injection of STZ (B+B_a_) which this results indicate the preventive effects of aqueous extract of *Berberis integerrima *Bge. root on liver injuries caused by diabetes.

Flavonoids and alkaloids have been shown to be potential antioxidants in the treatment of STZ induced oxidative stress in diabetic rats (44,45). The fruit, root and stem bark of barberry, contain various flavonoids and alkaloids which have antioxidant, anti-inflammatory and therapeutic properties (44,46). So antioxidant activity of barberry plant is probably related to phenolic, alkaloids and flavonoids compounds. Also antioxidant properties of barberry fruit and root have been proven in many studies (44,45). Aqueous extract of *Berberis integerrima *Bge. root may also act by either directly scavenging reactive oxygen metabolites due to the presence of various antioxidant compounds or by increasing the level of endogenous antioxidant molecules or enzymes.

## Conclusion

The present study demonstrated that *Berberis integerrimcan *Bge. play a prevention and role in complications of liver that caused by diabetes. This effects may be is due to its antioxidant properties.
